# Prediction of vaccine hesitancy based on social media traffic among Israeli parents using machine learning strategies

**DOI:** 10.1186/s13584-021-00486-6

**Published:** 2021-08-23

**Authors:** Shirly Bar-Lev, Shahar Reichman, Zohar Barnett-Itzhaki

**Affiliations:** 1 The Dror (Imri) Aloni Research Center for Health Informatics, Ruppin Academic Center, Emek Hefer, Israel; 2 School of Engineering, Ruppin Academic Center, Emek Hefer, Israel; 3 Coller School of Management, Tel-Aviv University, Tel-Aviv, Israel

**Keywords:** Childhood vaccination, Epidemiology, Social media, Machine learning, Public health

## Abstract

**Introduction:**

Vaccines have contributed to substantial reductions of morbidity and mortality from vaccine-preventable diseases, mainly in children. However, vaccine hesitancy was listed by the World Health Organization (WHO) in 2019 as one of the top ten threats to world health.

**Aim:**

To employ machine-learning strategies to assess how on-line content regarding vaccination affects vaccine hesitancy.

**Methods:**

We collected social media posts and responses from vaccination discussion groups and forums on leading social platforms, including Facebook and Tapuz (A user content website that contains blogs and forums). We investigated 65,603 records of children aged 0–6 years who are insured in Maccabi’s Health Maintenance Organization (HMO). We applied three machine learning algorithms (Logistic regression, Random forest and Neural networks) to predict vaccination among Israeli children, based on demographic and social media traffic.

**Results:**

Higher hesitancy was associated with more social media traffic, for most of the vaccinations. The addition of the social media traffic features improved the performances of most of the models.

However, for Rota virus, Hepatitis A and hepatitis B, the performances of all algorithms (with and without the social media features) were close to random (accuracy up to 0.63 and F1 up to 0.65). We found a negative association between on-line discussions and vaccination.

**Conclusions:**

There is an association between social media traffic and vaccine hesitancy. Policy makers are encouraged to perceive social media as a main channel of communication during health crises. Health officials and experts are encouraged to take part in social media discussions, and be equipped to readily provide the information, support and advice that the public is looking for, in order to optimize vaccination actions and to improve public health

**Supplementary Information:**

The online version contains supplementary material available at 10.1186/s13584-021-00486-6.

## Introduction

Vaccines have contributed to substantial reductions of morbidity and mortality from vaccine-preventable diseases, mainly in children [1]. Still, in 2019, vaccine hesitancy is listed by the World Health Organization (WHO) as one of the top ten threats to world health [2]. And so, vaccine compliance remains inconsistent and is therefore a growing concern [3]. Hesitancy can be defined as any manifestation between full acceptance of vaccines, and outright refusal of all vaccines [4] Kumar et al., claim that vaccine hesitancy can be caused by a complex interaction between socio-demographic factors (age, race, education, income); immune-specific characteristics (immunization requirements, vaccine-efficacy, vaccine safety), and sociological factors such as norms and attitudes. Hesitancy can be impacted by lack of information, access to misinformation, and is undermined by distrust of medical and government sources, which are sometimes seen as overplaying the risk or severity of disease and underplaying adverse side effects of vaccines [3, 5]. Since the relationship between on-line communication and vaccination hesitancy is complex and indirect, we need more than self-reports to assess it. To that end, we applied specialized machine learning tools that thus far had only been scantly applied to health research.

This study employs machine-learning strategies to assess the association between on-line content regarding vaccination and vaccine hesitancy. We collected a large amount of social media posts and responses that were posted in vaccination discussion groups and forums on leading social platforms, including Facebook and Tapuz (A user content website that contains blogs and forums). Our goal was to predict vaccination hesitancy by applying semantic analysis to vaccination related discourse in the social media. Specifically, we examined the predictive power of social media activity on aggregated vaccination activity between the years 2016–2018. The study is innovative in two respects: 1. It applies machine learning techniques to predict the effect of social media communication on vaccine behavior; 2. It is based on a ‘naturally occurring discourse’ rather than on subjective self-reports.

The study focuses on routine childhood vaccination programs for three reasons: (a) these vaccines are included in the National Health Insurance Law. Yet, they are not mandatory, thus requiring parents to take active steps to vaccine their children; (b) in Israel, routine childhood vaccinations are offered free of charge to all children at community-based child-health clinics for young children (birth to six years) and by school health services for schoolchildren (six to 15 years) [6]; and (c) much of the discourse about vaccination revolves around well-established immunization to well-understood childhood diseases. And so, this type of vaccines can suffer from the ‘curse of success,’ because their widespread administration has in many cases reduced incidence levels of the relevant diseases to near-zero.

The overall vaccination coverage rates reported in Israel are consistently high (97%), with the highest rates found among ultra-Orthodox Jews (98%) [7, 8]. On the other hand, recent studies point to a growing phenomenon of vaccine hesitancy, which has become more prevalent in Israel as in other countries during recent years [1, 7–9]. Studies indicate that the percentage of parents deviating from the recommended protocols amount to 7.5–9% [4, 10]. These studies indicate that for more than half of the parents, vaccination hesitancy is not caused by objective barriers of availability or cost but is a conscious decision [11].

In Israel, vaccination hesitancy is associated with higher education, affirming that hesitancy results from reflection and convictions, rather than ignorance [4]. In the ultra-Orthodox Jewish community, vaccination hesitancy is also explained by maternal academic education, having parents holding religious beliefs against vaccination, mistrusting the Ministry of Health, and perceiving the risk of vaccine preventable diseases as low [4, 8]. A recent study designed to evaluate current vaccination compliance rates among Israeli populations, implies that vaccination compliance, even among medically informed individuals, relies on a personal risk–benefit perception that may be influenced by misinformation regarding vaccine safety [3].

The sources of information regarding vaccination are changing with increased public exposure to the internet, social media, and online networks [9]. Their impact is especially strong during outbreaks, potentially influencing knowledge and attitudes. Social media has also been shown to be a major source of misinformation [9]. It is therefore not surprising that the Internet and social media play a significant role in the rise of fringe opinions that hampers public health’s efforts [2, 9, 10]. More concerning is the finding that a single access to an anti-vaccination site for just 5–10 min, increases the perception of the risks associated with the vaccines [9]. With regards to the COVID-19 vaccination, authors found that subjects with a hesitant or reluctant attitude toward vaccination used the mass media, the Internet, friends and relatives as their main sources of information on vaccination topics [9, 12]. People also tend to trust nontraditional sources of information more than official sources [13, 14].

While enlightening, these studies are based on self-reported data collected via surveys. A new trend of studies harnesses various data science and machine learning techniques to map the Internet and profile the voices dominating the Internet. Such studies have shown that people who use either Twitter or Facebook, or both, as sources of health information are more likely to have obtained the influenza vaccine than are people who do not [15]. Getman and his colleagues, for instance, built a searchable big-data platform of over 550 million posts from 50,000 media sources to study the influence of vaccine-hesitant content online, and showed that antivaccine content is uncommon online, as well as in mainstream media. Moreover, they showed that vaccine hesitant communities display strongly clustered linking behavior toward itself. Chan, Man-pui, Jamieson, and Albarracin (2020) suggest that despite these studies’ useful evidence, their findings are not based on longitudinal data and do not sufficiently combine individual and regional level data that are necessary to understand the relations between exposure to social media messages and behaviors [16, 17]. To address this void, this paper applies data science tools, particularly sentiment analysis tools and machine learning algorithms, to study the associations between social media discussions (posts and responses) and longitudinal data of childhood vaccination coverage in Israel.

These studies’ underlying assumption is that social media do not exist in a vacuum, and is reflective of the local context in which it is produced [16, 17]. Automated sentiment analyses, performed using supervised machine learning have been shown to provide strong and accurate results in estimating correlations between online activity around vaccination and coverage or outbreak data [18]. We can thus assume that online discussions are a good proxy for vaccine confidence in a country or region [18]. Our guiding research question is therefore: Are there any statistical associations between social media messages and vaccination hesitancy with regards to childhood vaccination in Israel between the years 2016–2018?

## Methods

### Data collection

The study is based on the analysis of 65,603 anonymized electronic medical records between the years 2014 and 2018, collected from Maccabi Healthcare Services (Maccabi) database. Maccabi’s Health Maintenance Organization (HMO) is the second largest HMO in Israel with over two million insured, and an annual growth of 36,000 insured in 2019. Maccabi’s clients are representative of the Israeli population, and reflect all demographic, ethnic, and socioeconomic groups and levels [14]. Permission to study the data was granted under the strict guidelines of the HMO’s Helsinki ethical committee. The database includes records from the years 2014–2018 with the following variables: child’s sex, child’s age, mother’s age, geographical residency (district), month of birth and socioeconomic status by income percentiles. We discerned six vaccinations, all of which comprise the recommended vaccination program by the Israeli Health Ministry for children aged 0–6. These vaccines are: Hepatitis B (2nd dose), Diphtheria, tetanus, and whooping cough (pertussis) (DTaP), Polio (IPV), Pneumococcal (PCV), Rotavirus (RV), and MMR.

The Israeli media 2020 report indicates that the social media platform Facebook is the most widely used social media site in Israel, and spans users with diverse socio-economic backgrounds (Orr, Baram-Tsabari, and Landsman, 2016). In Israel, there are about six million Facebook users. The users’ age distribution is: 18% in the range of 18–25 years old, 30% in the range of 25–34 years old, 18.6% in the range of 35–44, 12% in the range of, 45–54, and 11% in the age of 55 years old, or older. (https://datareportal.com/reports/digital-2020-israel). During 2014–2018, the rate of Internet use ranged between 75 to 83%, and Facebook use was stable.^1^

Posts were collected from three major sources: (1) A Facebook group “talking about vaccines”, that contained 54.7 K listed members; (b) A Facebook group “vaccines—an informed choice”, that contained 15.6 K listed members; and (c) A general forum named Tapuz -vaccination, that contained 4,000 members. These groups are public and open for all to join and participate. All three include both pro- and anti-vaccination messages. The data collection included the original post and the reactions to the post (like/dislike; share/comment) between 2013 and 2018. We counted the number of likes/dislikes, shares and comments each post received. In total, the data included 4707 posts from the Facebook group “talking about vaccination”, 4612 posts from the Facebook group “vaccines—an informed choice” and 244 from the Tapuz forum.

For each social media post, we first used Google Translate API to translate the posts’ text from Hebrew to English to allow the use of a robust sentiment analysis tool. We then calculated the post sentiment score (between − 1 to 1, where 0 represents neutral text) using a lexicon-based R package [15]. To account for the social engagement level (number of views, likes, shares and comments) to each post we also generated a weighted score similar to [19]:

$$W_{i} = \mathop \sum \limits_{i}^{n} \left( {log\left( {\left( {number\, of\,\alpha } \right)} \right) 1} \right) \left( {intensity\,of\,\alpha } \right)$$

where n = number of features, *α* = feature (views, likes, shares, comments).

**Fig. 1:**
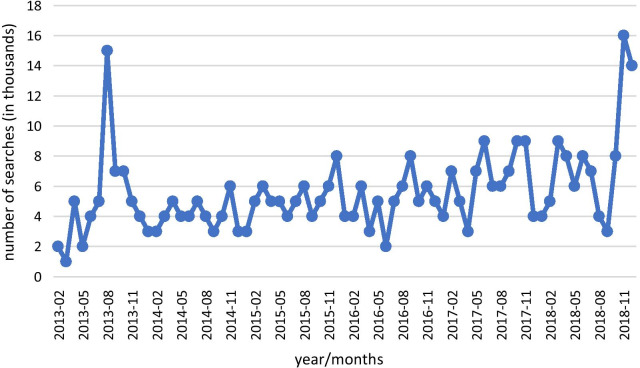
Internet searches for the term “vaccination” between 2013 and 2018 per month (Google trends)

We used the Python package pythrends to convert a collection of documents (*i.e.*, the texts extracted from Facebook) into a frequency matrix of token counts. Each extracted text was dated by month, so that these scores were aggregated to a monthly level score to align with the aggregated vaccination data. In addition, we studied the yearly search of the term “vaccines” between the years 2013–2018. As  Fig. 1   shows, there are small demonstrable differences in searching the word “vaccination” between 2013 and 2018. The models we built predict the rates of vaccination based on demographic features and the monthly on-line communication (Fig. 1, supplementary). It can be seen that during the winter months the search for “vaccinations” surges. On avarege, the level of searches for each year remains fairly similar.

### Predictive power assessment

To evaluate the out-of-sample predictive performance of on-line media activity, we used three different machine learning algorithms to predict vaccination among Israeli children (see below). The baseline model was created using authenticated demographic data derived from Maccabi HMO vaccination data. We then created an integrated model that includes the demographic features and the social media traffic features. Lastly, we compared the two models.

### Vaccination prediction models based on machine learning algorithms

First, we build a baseline models based on the data collected from Maccabi HMO. This model was designed to predict vaccination hesitancy as dependent on the mother’s demographic data. We then built other models which added the aggregated social media activity to show their predictive power in forecasting (cumulative) immunization decisions. These models were built to predict the vaccination hesitancy in a specific month as dependent both on the mother’s demographics and on the social media traffic at that specific month. The aggregation was performed by averaging the tone and sentiment across all posts on these sites at month *i* in our period of analysis.

We used three different machine learning algorithms to predict vaccination among Israeli children: Logistic regression, Random Forest and Neural Networks. Logistic Regression (LR) models the effect of a multi-class series of features (such as demographic features) and categorical output variables (such as vaccination). LR models were also used to assess the relative contribution of each of the features to the classification (*i.e.* which feature is more important to the prediction). Neural Networks (NN) are statistical models with a unique algorithmic structure that simulates the learning dynamics of the neural cells in the brain. NNs are based on a hierarchical node structure that receives multiple inputs, assigns each input a weight and outputs a decision based on the summation of these weighted data. Random Forest (RF) is an ensemble-based classification algorithm, based on generation of a large number of decision trees, each consists of a random subset of the original samples. The final classification is based on the majority vote of these sub models.

There was a significant imbalance between the classes: the numbers of vaccinated children were much higher than the non-vaccinated children (1.5 times in the case of Hepatitis A vaccination to 7.6 in case of DTaP-Hib-IPV). We therefore balanced the classes by a random reduction of samples from the bigger class.

As mentioned above, the baseline models included only the demographic features (sex, age, mother’s age, geographical residency (district), month of birth and socioeconomic status. We then generated the integrated models that included the social media traffic features (engagement and ton of the original post), in addition to the demographic features.

Categorical features such as district and sex were transformed to binary ones using the dummy variable approach. Numeric features were normalized using normal normalization (Z-score).

### Model evaluation

Data were randomly split into a training set (80% of the data) and a test set (20%), for calculating the algorithms’ performances metrics. Same training and test sets were used for all algorithms. Each model was run ten times (using ten different random splits). The presented performances are the average of the ten random splits.

We calculated the following performance metrics: accuracy ((True positive + True negative)/All population), error rate (1-accuracy), precision (True positive/(True positive + False positive)), recall (True positive/(True positive + False negative)), F1 score ((2 * Precision * Recall)/ (Precision + Recall)), and AUC (Area under curve of ROC curve).

## Results

### Vaccination hesitancy in Israel: 2014–2018

**Fig. 2:**
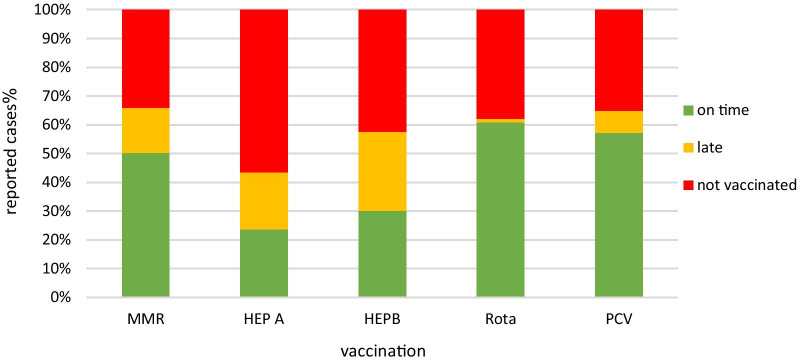
The percentage of vaccinated, late vaccinated, and not vaccinated participants between 2013 and 2018

Analyzing the numbers of children vaccinated between the years 2014–2018 shows that for most of the vaccines, most parents tend to vaccinate on time. However, relative to other vaccines, there is a relatively high rate of hesitancy in Hepatitis-A and Hepatitis-B vaccinations (see Fig. 2).

**Fig. 3:**
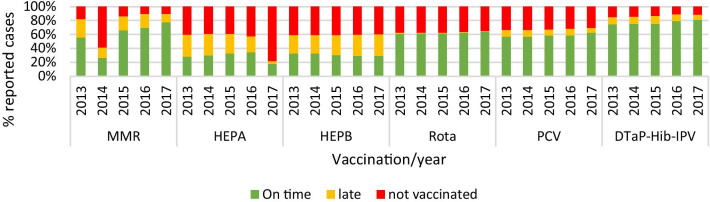
The rates of vaccination in the year 2013–2017 per vaccination type

Figure 3 shows the rate of on-time, delayed and non-vaccinated, per vaccination type, per year. Hepatitis-A and Hepatitis-B consistently show relatively high levels of vaccination hesitancy.

Next, we examined the associations between the number of children in the family and the vaccine hesitancy (late vaccination or no vaccination). We found that the higher the number of children in the family, the higher the vaccine hesitancy (Table 1).

**Table 1:** Correlations between number of children and vaccination hesitancy for each of the vaccination types

Vaccination	Not vaccinated/all correlated with # of child		Late/all vaccinated correlated with # of child	
	correlation coefficient	p-value	correlation coefficient	p-value
MMR	**0.44**	**0**	− **0.04**	**0.049**
HEP A	**0.293**	**0**	− **0.07**	**0**
HEP B	**0.05**	**0**	− **0.09**	**0**
PCV	− 0.01	0.5	− **0.1**	**0**
DTaP-Hib-IPV	− **0.03**	**0**	**0.01**	**0.002**

Bold value refer to statistically significant correlations

This finding is congruent with studies showing that birth order is associated with vaccination hesitancy. Firstborn children are known to have the highest odds of being immunized. As the birth order progresses the likelihood of vaccine utilization decreases significantly [20]. This finding has been found consistent even after controlling for other socio-demographic factors such as ethnicity and education, and suggests that the higher the number of siblings, the higher the likelihood of vaccine hesitancy. A possible explanation is that parents with many children have less resources/time to invest in non-acute health issues, such as vaccinations.

### Using machine learning tools to predict vaccination

In general, the addition of the social media traffic feature improved most of the models. However, for Rota virus, Hepatitis-A and hepatitis-B, the performances of all algorithms (with and without the social media features) were close to random (accuracy up to 0.63 and F1 up to 0.65).

### Using machine learning tools to predict MMR vaccination

The models based on the demographic features enable a good prediction of MMR vaccinations, with accuracy rates of 72%, F1 of 0.75, and AUC of 0.76 (Additional file 1: Table 1). Interestingly, the month of birth, particularly births in winter months (November to February), and the child birth’s year contributed to the prediction of vaccination more than other features. A recent study [1] concurs that delayed vaccinations are associated with season of birth. However, the authors do not offer an explanation. We can thus assume that children who are born in winter are more likely to be vaccinated on time due to their higher awareness of morbidities caused by winter viruses, and due to a general heightened exposure to vaccination campaigns.

In the case of MMR vaccination prediction, the addition of the social media traffic features did not improve the models’ performances. This can be explained by the fact that MMR vaccine is an uncontested vaccine perhaps due to emergence of measles and mumps outbreaks in Israel during the last decade [1]. Furthermore, MMR is considerably less delayed, perhaps because MMR is a single-dose vaccine not depending on timing of previous doses.

### Using machine learning tools to predict PCV vaccination

The models based on the demographic features yielded performances close to random. The addition of the social media traffic features significantly improved the models’ performances: with accuracy rates of 66%, F1 of 0.65, and AUC of 0.72 in the neural networks (Additional file 1: Table 2). The three features most contributing to the predictions were: (a) average of reactions, shares and likes, a month before the vaccination; (b) average of reactions, shares and likes, two months before the vaccination; and (c) score-weighted F1—the average of all engagement (using a separate weight for each engagement). Of note, the contributions of these features were significant but negative: more traffic on the social media means less vaccinations.

### Using machine learning tools to predict DTaP-Hib-IPV vaccination

The models based on the demographic features, yielded good performances: with accuracy rates of 0.68, F1 of 0.71 and AUC of 0.73 (Additional file 1: Table 3). Interestingly, the districts in which the children live, were the features most contributing to the prediction of vaccination: particularly the Sharon district, the southern district, the central district and the Jerusalem district.

The addition of the social media traffic features slightly improved the models’ performances: with accuracy rates of 73%, F1 of 0.73, and AUC of 0.79 in the random forest model. The three features most contributing to the predictions were: (a) average of reactions, shares and likes, a month before the vaccination; (b) average of reactions, shares and likes, two months before the vaccination; and (c) score-weighted F1—the average of all engagement (using a separate weight for each engagement). Again, the contributions of these features were significant be negative: more traffic on the social media means less vaccinations.

## Discussion

The data from the HMO’s registry confirm that most parents continue to vaccinate their children according to the Israeli Health Ministry’s recommended schedule. This can suggest a belief in the benefits of vaccination and trust in the advice from health care providers. However, for some of the vaccines, hesitancy and doubts regarding the instituted immunization schedule have become more common [16, 17, 19, 20]. We hypothesized that the tone of on-line discussions can be related to vaccine hesitancy on the aggregative level.

The semantic analysis of the on-line discussions supports the hypothesis that the volume of on-line discussion is associated with vaccination behavior. Although not investigated in this current study, we believe that negatively toned discussions can discourage vaccination. Our findings thus concur previous studies suggesting that on-line discussions can dissuade parents from vaccinating their children, or at least delay vaccination [8, 9]. One explanation is that on-line discussions inflate parents’ vaccination concerns in a way that discourages parents to comply with the official vaccination program. Another explanation can be related to individuals’ perceptions of how widespread (or consensually shared) certain beliefs about vaccination are [22]. Past research showed that participants in on-line forums who hold conservative opinions with regards to vaccination tend to overestimate the extent to which likeminded others share their beliefs (*i.e.*, judging their beliefs to be more common than they actually are), thereby exhibiting a “truly false consensus effect” [16]. By contrast, liberals may be prone to underestimate the extent to which likeminded others share their beliefs (*i.e.*, judging their beliefs to more distinctive than they actually are), thereby exhibiting “an illusion of uniqueness” [22]. With regards to health-related issues, the perceived prevalence of harmful behaviors—such as smoking tobacco, binge drinking, and illicit drug usage—not only affects the likelihood that a person will engage in that behavior, but the likelihood that people will use these perceptions to justify their behavior [23].

Thus, the perceived prevalence of one’s attitudes about vaccination, and their accuracy have broad-based implications for personal and public health outcomes [24]. We suggest that the on-line forums can create the effect of a “truly false consensus effect”. By presenting only one view as salient, these forums might give the impression that any other behavior negates the norm thus justifying harmful health related behavior. Consequently, for people who hold non-conservative attitudes towards vaccination, an intense and consistent exposure to information can contribute to a mindset that is distrusting of scientific recommendations.

Despite its contribution, our study has several limitations: (a) the features used in this analysis (that were supplied by Maccabi HMO) are quite limited. Addition of several important demographic and clinical features (*i.e.*: education, level of religiosity, background diseases etc.) could have significantly improved our models and could have improved our understanding; (b) The immaturity of sentiment analysis methods for texts in Hebrew limited our focus, and allowed an analysis based on the intensity of traffic, and only marginally on its tone (negative/positive). We expect that the further development of these tools will allow for a more nuanced analysis. Nevertheless, this study has several significant strengths: First, the data retrieved from Maccabi HMO is comprehensive and highly reliable. Second, we collected and analyzed thousands of posts from the most relevant sources related to the field of vaccinations, and third, we used advanced big data science approaches and machine learning tools to cope with this challenge.

By showing the possible relationship between the on-line discussions and vaccination behavior at the aggregative level, we also uncover the need to develop new methodologies that would allow a more nuanced understanding of how public opinion is formed with regards to childhood vaccination. This understanding is both timely and pressing as countries attempt to struggle with vaccine hesitancy with regards to the COVID 19 vaccine. Policy makers are encouraged to perceive social media as a main channel of communication during health crises. Social media should be acknowledged as voicing authentic dissident opinions and information that are worth addressing. Health officials and experts are encouraged to take part in social media discussions, and be equipped to readily provide the information, support and advice that the public is looking for, in order to optimize vaccination actions and to improve public health.

## Supplementary Information

**Additional file 1. Figure 1:** Social Media search in Israel between 2014–2018.

## Footnotes

1 https://data.worldbank.org/indicator/IT.NET.USER.ZS?locations=IL; https://gs.statcounter.com/social-media-stats/all/israel/#monthly-201401-201812
