# Supplementary material for: Prediction of vaccine hesitancy based on social media traffic among Israeli parents using machine learning strategies
Source: Isr J Health Policy Res. 2021 Aug 23;10:49. doi: 10.1186/s13584-021-00486-6 (PMC8381350; doi:10.1186/s13584-021-00486-6)

**Prediction of vaccine hesitancy based on social media traffic among Israeli Parents using Machine Learning Strategies – supplementary tables**

**Supplementary table S1: Performances of machine learning tools to predict MMRV vaccination, based on demographic, clinical features, and based on all features (demographic, clinical, and sentiment analysis features)**

|  | **Algorithm** | **accuracy** | **precision** | **recall** | **F1** | **Area under curve** |
| --- | --- | --- | --- | --- | --- | --- |
| All features | Neural network | 0.69 | 0.69 | 0.79 | 0.68 | 0.75 |
|  | Logistic regression | 0.7 | 0.7 | 0.79 | 0.7 | 0.76 |
|  | Random forest | 0.66 | 0.66 | 0.68 | 0.66 | 0.72 |
| Demographic feature | Neural network | 0.72 | 0.72 | 0.85 | 0.75 | 0.76 |
|  | Logistic regression | 0.66 | 0.68 | 0.63 | 0.65 | 0.68 |
|  | Random forest | 0.67 | 0.66 | 0.7 | 0.68 | 0.72 |

**Supplementary table S2: Performances of machine learning tools to predict PCV vaccination, based on demographic, clinical features, and based on all features (demographic, clinical, and sentiment analysis features)**

|  | **Algorithm** | **accuracy** | **precision** | **recall** | **F1** | **Area under curve** |
| --- | --- | --- | --- | --- | --- | --- |
| All features | Neural network | 0.66 | 0.68 | 0.66 | 0.65 | 0.72 |
|  | Logistic regression | 0.63 | 0.63 | 0.62 | 0.63 | 0.66 |
|  | Random forest | 0.56 | 0.56 | 0.55 | 0.56 | 0.58 |
| Demographic feature | Neural network | 0.54 | 0.53 | 0.76 | 0.62 | 0.55 |
|  | Logistic regression | 0.52 | 0.51 | 0.48 | 0.49 | 0.52 |
|  | Random forest | 0.4 | 0.4 | 0.44 | 0.42 | 0.34 |

**Supplementary table S3: Performances of machine learning tools to predict DTaP-Hib-IPV vaccination, based on demographic, clinical features, and based on all features (demographic, clinical, and sentiment analysis features)**

|  | **Algorithm** | **accuracy** | **precision** | **recall** | **F1** | **Area under curve** |
| --- | --- | --- | --- | --- | --- | --- |
| All features | Neural network | 0.64 | 0.61 | 0.8 | 0.6 | 0.72 |
|  | Logistic regression | 0.65 | 0.62 | 0.78 | 0.65 | 0.7 |
|  | Random forest | 0.73 | 0.74 | 0.72 | 0.73 | 0.79 |
| Demographic feature | Neural network | 0.66 | 0.62 | 0.82 | 0.71 | 0.71 |
|  | Logistic regression | 0.61 | 0.62 | 0.61 | 0.61 | 0.68 |
|  | Random forest | 0.68 | 0.68 | 0.67 | 0.67 | 0.73 |

Figure 1: An Analysis of Internet activity in Israel between 2013-2018


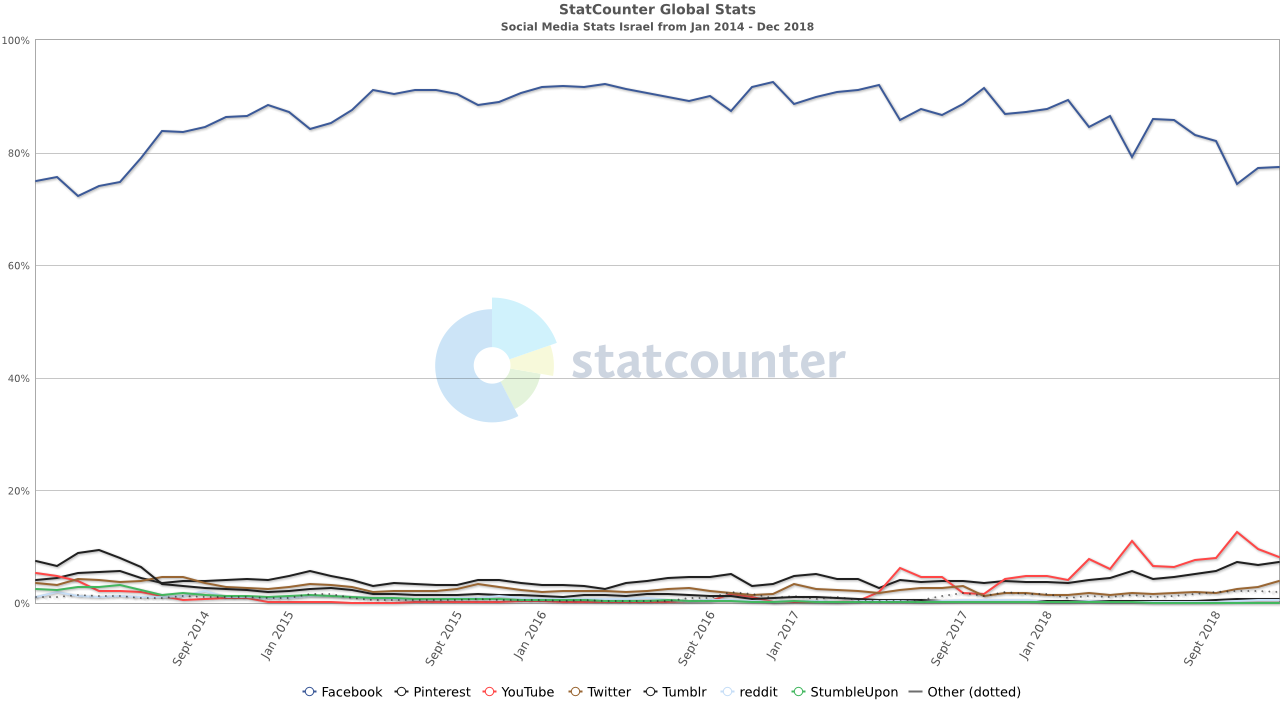

Supplement: Supplementary file 1 — Additional file 1. Figure 1: Social Media search in Israel between 2014–2018. [file 13584_2021_486_MOESM1_ESM.docx]
